# Anaphylaxis: first clinical presentation, subsequent referral practise, and suspected elicitor—an observational study

**DOI:** 10.1007/s11739-024-03589-5

**Published:** 2024-04-10

**Authors:** Julie Holst Gudichsen, Emil Aggerholm Bækdal, Frederik Bloch Jessen, Annmarie Touborg Lassen, Carsten Bindslev-Jensen, Charlotte G. Mortz, Søren Mikkelsen

**Affiliations:** 1 Department of Dermatology and Allergy Centre, Odense Research Center for Anaphylaxis, Odense University Hospital, University of Southern Denmark, Kløvervænget 15, 5000 Odense C, Denmark; 2 The Prehospital Research Unit, Region of Southern Denmark, Odense University Hospital, Odense C, Denmark; 3 Emergency Medicine Research Unit, University of Southern Denmark and Odense University Hospital, Odense C, Denmark

**Keywords:** Anaphylaxis, Prehospital, Emergency department, Referral for anaphylaxis

## Abstract

Anaphylaxis is an allergic manifestation characterised by rapid onset and progression. Rapid treatment may be challenging in patients with atypical symptoms or no previous history of anaphylaxis. This study aimed to describe the clinical prehospital presentation of first-time anaphylactic patients. To help target educational initiatives, we sought to identify which groups of medical professionals are most likely to encounter first-time anaphylactic patients and investigated the referral pattern for suspected anaphylactic patients for specialised treatment. A retrospective register-based study from the Region of Southern Denmark. Patients referred to the Allergy Centre, Odense University Hospital, from 2019 to 2021 were included. The medical records were manually reviewed for first contact with the emergency departments or the emergency medical service. 444 patients with suspected anaphylaxis were referred. 226 patients had grade 3–5 systemic allergic reactions as classified by the World Allergy Organisation; 90% had cutaneous symptoms, 63% symptoms from the central nervous system, 42% gastrointestinal symptoms, 40% cardiovascular symptoms, 36% had upper-airway symptoms, and 36% had lower-airway symptoms. Patients treated prehospitally had a significantly more severe degree of anaphylaxis than patients only treated within the hospital. More than half of the patients with suspected anaphylaxis were referred to the Allergy Centre from the emergency departments. Patients with allergies progressing to severe anaphylaxis most often are treated prehospitally before transport to emergency departments. From the emergency departments, they are referred to the allergy centre. Education concerning the immediate treatment of severe anaphylaxis should primarily be targeted towards prehospital care providers.

## Introduction

Anaphylaxis is the most severe form of allergy and fast diagnosis and treatment is therefore crucial [1–3]. The diagnosis is challenging if the patient has an atypical presentation of symptoms (e.g., without the involvement of the skin) and, in particular, if the patient has no previous history of anaphylaxis [1, 2, 4–6]

A recent study estimated that the incidence rate of first-time anaphylaxis is 26.8 cases per 100,000 person-years in children and 40.4 cases per 100,000 person-years in adults [7]. The most common elicitors of anaphylaxis are reportedly prescription drugs and over-the-counter medicine (41.1%), insect venom (27.4%), and food (20.6%); the latter being more common among children than in adults (8). In 10.5% of cases, an elicitor may not be identified even after a thorough diagnostic workup in a highly specialised allergy centre [8].

Anaphylaxis is a clinical diagnosis. Because of the rapid onset immediate treatment with intramuscular adrenaline is necessary [1, 2, 5, 9]. A study investigating the treatment of anaphylaxis by prehospital anaesthesiologists showed that even in cases with severe anaphylaxis, most patients were not treated with adrenaline but were instead treated with intravenous antihistamines and glucocorticoids alone [10]. This tendency in the treatment of patients with anaphylaxis has also been found in emergency departments (EDs) [8, 11–13].

This apparent lack of coherence with treatment protocols both prehospitally and in the EDs may be detrimental to patients and calls for further educational initiatives. To target these educational initiatives we sought to elucidate how anaphylactic patients present themselves clinically at the first contact with the health care system. Further, we sought to map which care provider in the acute health care system first recognizes anaphylaxis and refer the patients to specialised assessment.

This study thus aimed to investigate (1) the clinical presentation (including vital parameters) of allergic patients the first time they presented with symptoms leading the clinician to suspect anaphylaxis prehospitally or in the emergency departments, and (2) the primary contact point with the health care system for these patients.

## Methods

This is a retrospective register-based study based on data from the Allergy Centre at Odense University Hospital, the EDs, and the emergency medical system (EMS) in the Region of Southern Denmark covering the period January 1, 2019, to December 31, 2021.

### System setting

The Danish EMS is a tax-funded three-tiered system. The primary prehospital resource, the first tier, is an ambulance manned by two paramedics. The second tier consists of a paramedic in a rapid-response car. A third tier consists of an anaesthesiologist in either a ground-based unit or a helicopter-based unit [14, 15]. In the Region of Southern Denmark, in approximately 25% of the cases, an anaesthesiologist is dispatched along with the ambulance [16]. Following prehospital treatment, patients are usually transported to an ED of which there are six in the Region of Southern Denmark. The EDs are manned by physicians specialised in Emergency Medicine or Internal Medicine.

The Allergy Centre at Odense University Hospital is a highly specialised department that performs diagnostic evaluation and treatment of patients with suspected allergies. The diagnostic process includes obtaining the medical history surrounding the suspected allergic reaction, allergy tests, and subsequent allergen challenges. All patients from the Region of Southern Denmark (population 1.28 million) can be referred to the Allergy Centre following an anaphylactic episode or when an allergy is suspected.

All Danish citizens are assigned a unique identification number, the Civil Personal Register number (CPR number), that allows for unique patient identification [17]. The prehospital medical record system used in Denmark consists of one common national database into which emergency medical technicians, paramedics, and prehospital physicians enter the patients´ vital parameters, the examinations performed, and the treatments given [15]. At the ED and Allergy Centre, one identical in-hospital electronic medical record system is used.

### Participants

The patients included in this study were adult patients (≥ 18 years) with a first-time referral to the Allergy Centre for a suspected anaphylactic reaction. We used the World Health Organisation´s International Statistical Classification of Diseases and Related Health Problems 10th Revision (ICD-10) to identify cases [18]. The following ICD-10 diagnosis codes were used: T78.0, T78.0A, T78.2, T63.4F, T63.4G, and T88.6 (Anaphylactic shock due to adverse food reaction; Anaphylactic shock due to food allergy; Anaphylactic shock, unspecified; Anaphylactic Shock venom; Systemic allergic reaction venom; Anaphylactic shock due to adverse effect of correct drug or medicament properly administered).

Patients were excluded if the data containing information regarding the allergic incident originated from hospitals outside the Region of Southern Denmark, or if the patients had failed to show up at the planned consultation at the Allergy Centre. If a patient appeared more than once in the study period, only the first allergic reaction and subsequent visit to the Allergy Centre was included.

### Data collection

The database containing patients with a first-time referral to the Allergy Centre was used with the patient´s unique identifier, the CPR number, as the basic patient identifier [17]. The study population was assessed for contacts with the emergency medical system and the emergency department by manual scrutiny of the prehospital medical records and the in-hospital electronic medical records. In-hospital medical records from the first visit to the Allergy Centre were assessed and the following was registered:

Sex, age, referring physician, the suspected elicitor, and the ICD-10 diagnosis.

In stratifying the severity of anaphylaxis, the World Allergy Organisation (WAO) grading system [2] was used. This grading system is based on the registration of symptoms from the central nervous system, the cardiovascular system, the gastrointestinal system, the upper airway, the lower airway, cutaneous symptoms, conjunctival symptoms, and/or other symptoms. Anaphylaxis is corresponding to a WAO grade 3–5 [2]. When retrospectively estimating the WAO grade, only the specific WAO-relevant grading symptoms were used [2]. However, all symptoms and findings relevant to allergies recorded in the medical records were registered.

If contact with the EMS and/or the ED occurred in conjunction with the anaphylactic episode, the prehospital medical record and the in-hospital medical records were reviewed. The following variables were registered:

The first measured vital parameters (systolic blood pressure, respiratory rate, pulse rate, oxygen saturation, and temperature), any symptoms from the central nervous system, the cardiovascular system, the gastrointestinal system, the upper airway, the lower airway, any cutaneous symptoms or conjunctival symptoms, and/or other symptoms related to anaphylaxis.

All symptoms or findings possibly relevant to the diagnosis of anaphylaxis were registered. Symptom registration included (but was not limited to) those mentioned in the WAO grading system. The WAO grade, however, was estimated using only WAO-relevant symptoms and vital parameters [2].

### Statistical analysis

Data are presented as proportions, medians and quartiles (where appropriate). All data were analysed using non-parametric statistics. For binary exposure and outcome, Pearson’s chi-squared test was used. Kruskal–Wallis test was applied to determine if there were statistically significant differences between two or more groups of an independent variable. The Dunn test was applied to test for individual differences between subgroups. Differences were considered significant when *p* < 0.05. Data were analyzed using STATA 18 BE (StataCorp, College Station, Texas, USA).

### Ethics

The study was approved by the Danish Data Protection Agency (Journal no. 21/6778). Furthermore, the Director of the Odense University Hospital approved the study as a quality assurance study. The study was further approved as a quality assurance study by the Prehospital Director of the Region of Southern Denmark (Journal no. 21/27363).

## Results

**Fig. 1:**
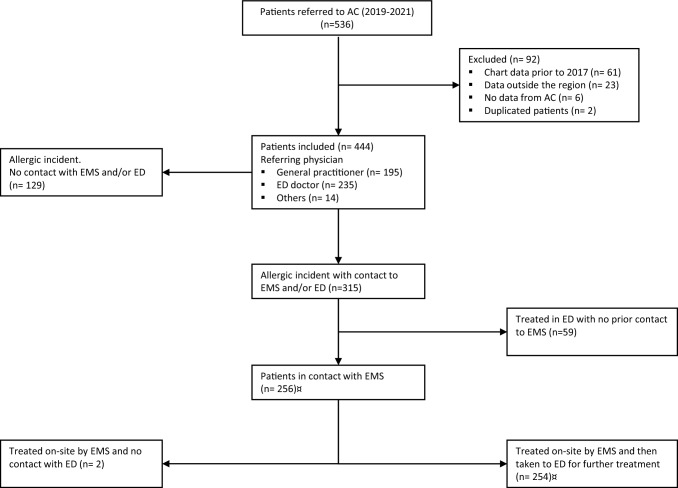
Flowchart showing the number of patients in each stage of the study, reasons for exclusion, and referring physician 12 patients had missing EMS medical records. *AC* Allergy Centre. *GP* general practitioner. *ED* emergency department. *EMS* emergency medical system

A total of 536 adult patients had a first-time referral to the Allergy Centre with a suspected anaphylactic reaction during the period 1 January 2019–31 December 2021. Of the 536 patients, 92 were excluded. Thus, 444 patients fulfilled the inclusion criteria. For the distribution of patients in the study, an overview of excluded patients, and the referring physician, see Fig. 1.

The median age was 53.5 years at the time of the allergic incident (quartiles: 41.25–66) and 201 (45.3%) were women; 243 (54.7%) were men.

### Clinical prehospital presentation

Of the 444 patients included in this study, 256 (57.7%) had been in contact with the EMS. The prehospital records were available in 244 of these cases. The measured vital parameters and the organ system manifestations are presented in Table 1. Of the 244 patients with available EMS records, 115 (47.1%) had symptoms corresponding to a WAO score of 3–5, with 62 (25.4%) being graded as WAO 5. Cutaneous symptoms were observed in 223 (91.4%) of all cases. The second-most frequent manifestations were symptoms from the central nervous system (*n* = 94, 38.5%) and the cardiovascular system (*n* = 54, 22.1%).

**Table 1:** Patient characteristics upon first prehospital presentation (*n* = 244)

EMS WAO grade	0–2	3	4	5	Total	*p*-value
Number of patients	129	26	27	62	244	
Vital parameters *						
Systolic blood pressure	143.5 (127–155)	142.5 (120.75–158.75)	137 (115.5–154)	88 (72.75–121)	136 (110.5–152.5)	0.015
(No. of observations)	*n* = 128	*n* = 24	*n* = 26	*n* = 62	*n* = 240	
Heart rate	88 (74–98.25)	89 (80.5–95)	89 (74.5–101.5)	80 (67.5–92.25)	85 (72–97)	0.884
(No. of observations)	*n* = 126	*n* = 25	*n* = 26	*n* = 62	*n* = 239	
Oxygen saturation	98 (96–99)	98 (95.75–100)	97.5 (95–100)	95 (92–96)	97 (94.5–98.5)	0.0001
(No. of observations)	*n* = 127	*n* = 26	*n* = 26	*n* = 61	*n* = 240	
Respiratory rate	18 (16–20)	20 (18–24)	20 (18–25)	20 (18–22)	18 (16–20)	0.0001
(No. of observations)	*n* = 115	*n* = 22	*n* = 22	*n* = 52	*n* = 211	
Clinical symptoms (%)						
Central nervous system	24 (18.6)	4 (15.4)	6 (22.2)	60 (96.8)	94 (38.5)	
Gastrointestinal tract	2 (1.6)	7 (26.9)	1 (3.7)	28 (45.2)	38 (15.6)	
Cardiovascular	11 (8.5)	6 (23.1)	11 (40.7)	26 (41.9)	54 (22.1)	
Upper airways	15 (11.6)	4 (15.3)	27 (100)	15 (24.2)	61 (25)	
Lower airways	9 (7)	18 (69.2)	6 (22.2)	6 (9.7)	39 (16)	
Cutaneous	125 (96.9)	24 (92.3)	23 (85.2)	51 (82.3)	223 (91.4)	
Conjunctival	26 (20.2)	4 (15.4)	4 (14.8)	3 (4.8)	37 (15.2)	
Other	16 (12.4)	2 (7.7)	1 (3.7)	3 (4.8)	22 (9)	

*n* number of patients with registered vital parameters, *median (quartiles)

For the distribution of the predominant symptoms in patients with anaphylaxis and the corresponding vital parameters, see Table 1 where the patients are stratified according to the severity of the allergic reaction (WAO grade 0–2, and WAO grades 3, 4, and 5).

### Clinical presentation at the emergency department

Of the 444 patients included in the study following referral to the AC, 313 (70.5%) patients were treated at the ED. Upon arrival at the ED, 75 (24.0%) of these patients presented with symptoms corresponding to a WAO grading of 3–5, 40 (12.8%) of those fulfilling criteria for WAO 5, severe anaphylaxis. Cutaneous symptoms were observed in 253 (80.8%) of all cases. The second-most frequent manifestations were symptoms from the central nervous system (*n* = 64, 20.4%) and the cardiovascular system (*n* = 58, 18.5%). The measured vital parameters and distribution of organ system involvement are shown in Table 2. Patients are stratified according to the severity of the allergic reaction (WAO grade 0–2, and WAO grades 3, 4, and 5).

**Table 2:** Patient characteristics upon first presentation at the Emergency Department (*n* = 313)

ED WAO grade	0–2	3	4	5	Total	*p*-value
Number of patients	238	26	9	40	313	
Vital parameters *						
Systolic blood pressure	135 (123–150)	131 (115–136)	146 (132–146)	85 (74–92)	132 (116–147)	0.241
(No. of observations)	*n* = 225	*n* = 23	*n* = 9	*n* = 39	*n* = 296	
Heart rate	79 (70–90)	82 (70.75–88.25)	91 (73.25–104)	74 (65–84)	79 (70–90)	0.536
(No. of observations)	*n* = 225	*n* = 24	*n* = 8	*n* = 35	*n* = 292	
Oxygen saturation	98 (97–100)	99 (97–100)	99 (97–100)	97 (95–100)	98 (97–100)	0.316
(No. of observations)	*n* = 228	*n* = 24	*n* = 9	*n* = 37	*n* = 298	
Respiratory rate	17 (16–20)	17 (16–20.75)	18 (16–20)	18 (16–20)	17 (16–20)	0.543
(No. of observations)	*n* = 207	*n* = 22	*n* = 7	*n* = 33	*n* = 269	
Clinical symptoms (%)						
Central nervous system	30 (12.6)	9 (34.6)	2 (22.2)	23 (57.5)	64 (20.6)	
Gastrointestinal tract	6 (2.5)	15 (57.7)	1 (11.1)	14 (35)	36 (11.5)	
Cardiovascular	18 (7.6)	4 (15.4)	4 (44.4)	32 (80)	58 (18.5)	
Upper airways	17 (7.1)	5 (19.2)	9 (100)	11 (27.5)	42 (13.5)	
Lower airways	2 (0.8)	13 (50)	1 (11.1)	5 (12.5)	21 (6.8)	
Cutaneous	184 (77.3)	26 (100)	7 (77.7)	36 (90)	253 (75.6)	
Conjunctival	38 (16)	6 (23)	1 (11.1)	5 (12.5)	50 (16)	
Other	12 (5)	1 (3.8)	0 (0)	5 (12.5)	18 (5.8)	

n = number of patients with registered vital parameters *median (quartiles)

Table 2* near here.*

Of the patients seen in the ED, 59 (18.8%) did not have any preceding contact with the EMS. These patients either presented at the hospital on their own accord (*n* = 27), were referred by an out-of-hours general practitioner (*n* = 18), a general practitioner (*n* = 10), or were referred from elsewhere (*n* = 4). Twenty-three (39.0%) of these patients had a WAO grade of 3 or above and 15 (25.4%) had a WAO grade of 5 at the arrival to the ED.

### Patients referred to the allergy centre without any previous contact with the EMS or the ED

129 patients had no contact with the EMS or the ED before being referred to the Allergy Centre. Thirty-five of these (27.1%) were assessed as having reacted corresponding to a WAO grade 3 or above. Among these 129 patients, 84 (65.1%) were referred to the Allergy Centre with insect venom as the suspected elicitor, 24 (18.6%) with suspected food allergy, 10 (7.8%) with suspected drug allergy, 8 (6.2%) with unknown elicitor, and 2 (1.6%) patients with suspicion of an exercise-induced allergic reaction.

### Elicitors of anaphylaxis, the ensuing clinical presentation, and referral pattern

**Fig. 2:**
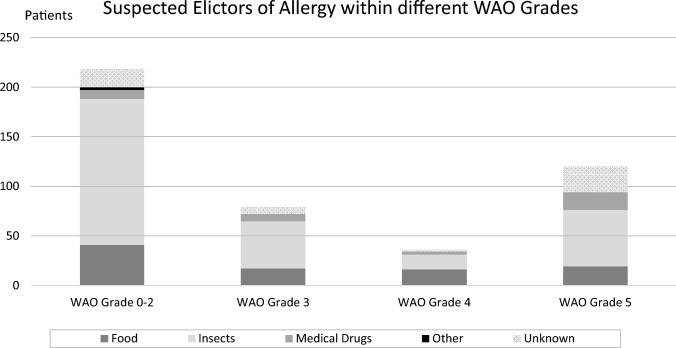
Graphical depiction of elicitors of anaphylaxis and the accompanying degree of anaphylaxis according to the World Allergy Organization classification system (WAO grade)

Of the 444 patients included in this study, 226 patients (50.9%) were found to have reacted consistent with anaphylaxis with an estimated WAO 3 grade or above. In the majority of these cases, the suspected elicitor was insect venoms. Across all WAO grades (0–5), foods were the suspected elicitor in 93 reactions. Of these, 52 had a WAO score of 3–5 (19 patients with severe anaphylaxis). Insect venoms were suspected to have been the eliciting agents in 267 reactions. Of these, 120 had a WAO score of 3 to 5 (57 of these patients with severe anaphylaxis). Finally, 37 patients had medical drugs (prescription drugs or over-the-counter drugs) as the suspected elicitor; 28 of these had a WAO score of 3 to 5 (57 patients with severe anaphylaxis). For a graphical depiction of the suspected elicitors of anaphylaxis and the accompanying degree of anaphylaxis, see Fig. 2.

The EDs were involved in the treatment of 191 (84.5%) of the 226 anaphylactic patients, and the EMS in 155 (68.6%) of the anaphylactic patients. Among patients with a confirmed anaphylactic reaction, a cutaneous manifestation was the most frequently observed symptom (*n* = 203, 89.8%), followed by symptoms from the central nervous system (*n* = 142, 62.8%) and the gastrointestinal system (*n* = 94, 41.6%).

The suspected elicitors, the clinical symptoms, and the referring physician are presented in Table 3. Patients are stratified according to anaphylaxis or not and sub-stratified according to their WAO grades.

**Table 3:** An overview of all included patients, elicitors, symptoms, and referring physician*

		Not anaphylaxis				Anaphylaxis		
WAO grade assessed by AC	0	1	2	Total	3	4	5	Total
	*n* (%)				*n* (%)			
Total Number of patients	15	104	99	218	79	36	111	226
Suspected elicitor								
Food	0 (0)	21 (20.2)	19 (19.2)	41 (18.8)	17 (21.5)	16 (44.4)	19 (17.1)	52 (23.0)
Insect	13 (86.7)	68 (65.4)	66 (66.7)	147 (67.4)	48 (60.8)	15 (41.7)	57 (51.4)	120 (53.1)
Medical drugs	0 (0)	5 (54.8	4 (4.0)	9 (4.1)	7 (8.9)	3 (8.3)	18 (16.2)	28 (12.4)
Other	1 (6.7)	1 (1.0)	1 (1.0)	3 (1.4)	0 (0.0)	0 (0.0)	0 (0.0)	0 (0.0)
Unknown	1 (6.7)	9 (8.7)	8 (8.1)	18 (8.3)	7 (8.9)	2 (5.6)	17 (15.3)	26 (12.4)
Clinical symptoms, *n* (%)								
Central nervous system	2 (13.3)	19 (18.3)	37 (37.4)	58 (26.6)	36 (45.6)	12 (33.3)	94 (84.7)	142 (62.8)
Gastrointestinal tract	0 (0)	4 (3.8)	5 (5.1)	9 (4.1)	38 (48.1)	10 (27.7)	46 (41.4)	94 (41.6)
Cardiovascular	4 (26.7)	7 (6.7)	22 (22.2)	33 (15.1)	19 (24.1)	7 (19.4)	65 (58.6)	91 (40.3)
Upper airways	1 (6.7)	4 (3.8)	28 (28.3)	33 (15.1)	19 (24.1)	34 (94.4)	29 (26.1)	82 (36.3)
Lower airways	0 (0)	6 (5.8)	5 (5.1)	11 (5.0)	39 (49.4)	14 (38.9)	29 (26.1)	82 (36.3)
Cutaneous	10 (66.7)	97 (93.2)	98 (99.0)	205 (94.0)	75 (94.9)	32 (8.9)	96 (86.5)	203 (89.8)
Conjunctival	1 (6.7)	1 (1.0)	39 (39.4)	41 (18.8)	18 (22.8)	7 (19.4)	22 (19.8)	47 (20.8)
Other	1 (6.7)	3 (2.9)	14 (14.1)	18 (8.3)	10 (12.7)	4 (11.1)	14 (12.6)	28 (12.4)
Referring physician, n (%)								
General practitioner	11 (73.3)	60 (57.7)	43 (43.4)	114 (52.3)	36 (45.6)	11 (30.6)	34 (30.6)	81 (35.8)
ED doctor	4 (26.7)	40 (38.5)	56 (56.6)	100 (45.9)	41 (51.9)	24 (66.7)	70 (63.1)	135 (59.7)
Other	0 (0)	4 (3.8)	0 (0.0)	4 (1.8)	2 (2.5)	1 (2.8)	7 (6.3)	10 (4.4)

Two patients were treated and subsequently released prehospitally by the EMS without any ensuing contact with the ED. Both patients were described with only mild local swelling after being stung by a wasp. Following a full diagnostic workup at the Allergy Centre, the patients were assessed as having had a WAO grade of 0 and the suspicion of anaphylaxis was thus rejected.

Of the 444 included patients, 235 (52.9%) were referred to the Allergy Centre from the ED, 195 (43.9%) by a general practitioner (GP), and 14 (3.2%) were referred by others (Fig. 1). Of the 226 patients who had experienced a reaction consistent with WAO 3 anaphylaxis or above, 135 (59.7%) were referred by the ED, a GP referred 81 patients (35.8%), and 10 patients (4.4%) were referred from elsewhere (Table 3). A significantly higher portion of patients had confirmed anaphylaxis when referred from the ED, compared to those referred from a GP (*p* = 0.001).

## Discussion

In this study on anaphylactic patients in the Region of Southern Denmark, we found that the first contact with the health care system for patients with anaphylaxis most often takes place prehospitally where the patients are treated by the EMS. These patients are in almost every case transported to the EDs from which the majority of referrals to the Allergy Centre take place. Furthermore, those referred to the Allergy Centre from the ED with the initial contact with the health care system taking place prehospitally had more severe reactions than those without EMS contact.

Only a few studies have evaluated the referral pattern of possible anaphylactic patients for diagnostic work-up by specialists in allergy.

One research letter [19] investigated the number of patients with anaphylaxis referred from EDs to an allergologist before and after an intervention consisting of the establishment of referral guidelines implemented in the EDs. The letter reported that no patients were referred before the establishment of referral guidelines while 44% were referred after this intervention, suggesting that clear guidelines may assist in the referral of patients. In our study, 64 patients were treated for anaphylaxis in the EDs but were not subsequently referred to the Allergy Centre by a physician from the ED but rather by their own GP. This may be due to the treating physician at the ED either forgetting to refer the patient or having estimated that the allergic reaction was not severe enough to be anaphylaxis. On the other hand, the treating physician at the ED may have written in the discharge summary (which in Denmark is sent to the patient’s GP) that a referral to the Allergy Centre should be made. The present study, however, did not review individual discharge summaries or examine local guidelines concerning the responsibility for referral on either the ED physician or the GP.

The clinical presentation of patients with anaphylaxis has previously been described in the literature. The World Allergy Organisation [1, 2] estimates that cutaneous symptoms are present in 80–90% of cases. This estimate is in line with other recent studies [20–22]. The symptoms may vary, depending on the patient´s age, the eliciting agent, and the severity of anaphylaxis [1, 2, 20]. A recent prospective Japanese study found cutaneous involvement in 98.3% of anaphylactic cases treated at the EDs, followed by respiratory symptoms (82.1%), and gastrointestinal symptoms (49.7%) [21]. This was also reported in a Danish prospective study [7] where the most common symptoms in anaphylactic patients presenting to the ED were cutaneous symptoms (93%), respiratory symptoms (79%), gastrointestinal symptoms (61%), cardiovascular symptoms (60%), and central nervous system (47%). Another multi-centre study based on data from 8,465 medical records found that the presence of cutaneous symptoms in anaphylaxis ranged from 76.8 to 89.3%, while cardiovascular symptoms ranged from 66.5 to 80.4% depending on the patient´s age [20]. These results align somewhat with the findings in our study, matching the prevalence of cutaneous and gastrointestinal symptoms, but suggest that an underreporting of cardiovascular and respiratory symptoms has taken place in our study. This might, however, be due to differences in symptom registration between studies. We registered the respiratory symptoms in two categories, upper airway symptoms and lower airway symptoms, whereas some studies register these symptoms as a combined entity [7, 20, 21].

Furthermore, we registered all symptoms, both the objective findings and the reported subjective symptoms. For instance, symptoms like ‘headache’ or ‘dizziness’ were included under ‘central nervous system’ symptoms. These are not included in the WAO grading system but are included in other studies describing the clinical presentation of anaphylactic patients [22, 23]. In our study, a higher proportion of patients with anaphylaxis also experienced symptoms from the central nervous system compared to those with non-allergic reactions (grade 0) or milder allergic symptoms (grade 1–2) but no anaphylaxis (WAO grade 0–2). This is consistent with other studies describing symptoms from the central nervous system as early signs of hypotension and harbingers of other severe reactions [24, 25].

The patients seen by both the EMS and ED had a significant improvement in WAO grade from the first prehospitally encounter to arrival at the ED. This might be caused by the medical treatment administered prehospitally or simply caused by the natural trajectory of anaphylaxis. Our study did, however, not investigate the treatment administered and further research is needed to clarify the cause of the improvements in WAO grade and vital parameters.

### Elicitors of anaphylaxis

The topic eliciting agents of anaphylaxis has been studied previously [20]. It has thus been reported that 51.2% of anaphylactic patients reacted to insect venom, 22.8% reacted to drugs, and 17.3% reacted to foods. This differs from a Danish study in both adults and children where drugs were the most common elicitor, followed by insect venom, and food [8]. Our study found that insect venom was the most commonly suspected eliciting agent accounting for 53.1% of anaphylactic reactions. The two studies cited above [8, 20] investigated confirmed elicitors whereas we in our study only registered the suspected elicitor. Furthermore, the present study only included adults while the former Danish study also included children. However, even though prescription drugs or over-the-counter medicines only accounted for 28 of the 226 cases of anaphylaxis in our study, 64.2% of reactions where drugs caused the anaphylaxis were severe anaphylactic reactions (WAO grade 5). This proportion of severity is found in other studies as well [26]. A study on 2,458 deaths caused by anaphylaxis reported that prescription drugs or over-the-counter medicine were responsible for most deaths (58.8%) [27]. Similar findings were reported in another study, where prescription drugs or over-the-counter medicine were accountable for more than 50% of fatal anaphylactic cases [28]. Our findings support these results and suggest that a high level of alertness is necessary if the suspected elicitor is prescription drugs or over-the-counter medicines.

### Recognition of anaphylaxis

Several studies [6, 29–31] describe anaphylaxis as ‘underdiagnosed’. Two multi-centre studies reviewed ED charts from North America for patients with an allergic reaction to either venom or food [32, 33]. The studies reported that of 678 patients diagnosed with an allergic reaction to food, 51% reacted severely enough to be categorised as anaphylaxis. Likewise, 617 patients were diagnosed to have an allergic reaction caused by insect venoms; 31% of these reacting severely enough to support a diagnosis of anaphylaxis. In these studies, anaphylaxis was not diagnosed initially because of an underestimation of the severity of the reaction. Another study investigated paramedics’ ability to recognise and treat anaphylaxis [11]. Paramedics were presented with two cases; a ‘classical’ anaphylactic reaction with skin symptoms and one without skin symptoms. Only 2.9% of paramedics listed anaphylaxis as a possible diagnosis if no cutaneous symptoms were included in the case. In this study, an underdiagnosing of anaphylaxis was thus demonstrated. In our study, 84% of the anaphylactic patients were seen by the EDs or EMS. Our finding that the patients with more severe cases of anaphylaxis first had contact with the prehospital care providers underscores that particular educational efforts in recognising anaphylaxis may be relevant in the prehospital care sector.

### Limitations and strengths of the study

A major limitation of this study is the retrospective design. The severity of anaphylaxis in each patient was classified retrospectively based on the documented presence of any symptoms relevant to the WAO classification and the first measured vital parameters recorded in the prehospital and the in-hospital medical records. Some symptoms or measurements may not have been registered in the patient’s medical records. Furthermore, in emergency cases, the first measurements of vital parameters may have been postponed till after the initial treatment. In patients with no contact with the EMS and/or ED before the referral to the Allergic Centre, our estimation of the WAO grade was solely based on the in-hospital records from the diagnostic work-up at the Allergy Centre containing the patient’s description of the episode. This recall bias may affect the validity of the described symptoms.

A further limitation is that our study was conducted in one single health region in Denmark, comprising one-sixth of the Danish population. As such, our results may not be generalizable to other healthcare systems in other settings.

The strengths of this study include the high number of patients included in this study. Furthermore, the unique patient identifier, the CPR number [16] ensures a very low number of patients lost to follow-up.

## Conclusion

The first encounter with the health care system for patients with severe anaphylaxis most often is with the emergency medical system. Educational initiatives should be targeted the prehospital care provider. The physicians manning the EDs and the general practitioners, however, should be aware of anaphylaxis per se, as the patients´ subsequent diagnostic work-up at the Allergy Centres is dependent on their identification of cases of anaphylaxis.

## Data Availability

Anonymised data are available from the corresponding author on reasonable request.

## Abbreviations

CPR: Civil personal register
ED: Emergency department
EMS: Emergency medical services
ICD-10: World Health Organisation´s International Statistical Classification of Diseases and Related Health Problems 10th Revision
WAO: World Allergy Organisation
